# Myeloperoxidase Deficiency: A Rare Case

**DOI:** 10.7759/cureus.63596

**Published:** 2024-07-01

**Authors:** Safa Mousavi, Mohammad Hossein Hosseini, Sadra Sarandili, Babak Nejati

**Affiliations:** 1 Public Health, California State University, Fresno, USA; 2 Hematology and Oncology, Tabriz University of Medical Sciences, Tabriz, IRN; 3 Health Sciences, Curtin Medical School, Perth, AUS

**Keywords:** primary phagocyte disorder, neutrophil dysfunction, myeloperoxidase deficiency, enzymatic deficiency, case report

## Abstract

Myeloperoxidase (MPO) is found in the lysosomes of monocytes and neutrophils, serving as a crucial component in the elimination of infections through the process of phagocytosis via neutrophils. Consequently, individuals with MPO deficiency exhibit a significantly heightened susceptibility to serious infections and chronic inflammatory diseases. In a clinical case, a 37-year-old Iranian woman presented with a chronic history of bacterial and fungal infections dating back to her childhood. She has no family history of similar diseases and has used antibiotics and antifungal medications. A comprehensive clinical assessment revealed that she is well-nourished and without acute distress, neurological symptoms, or cutaneous manifestations. A complete blood count (CBC) with differential white blood cell (WBC) count showed a decreased number of neutrophils despite normal WBC counts, and peripheral blood smear (PBS) revealed reduced neutrophil granulation, abnormal neutrophil morphology, decreased chromatin condensation, and cytoplasmic hypogranulation. So, the patient was diagnosed with MPO deficiency, a rare condition requiring early diagnosis and management.

## Introduction

Myeloperoxidase (MPO) is a heme-containing protein present in the granules of neutrophils and the lysosomes of monocytes [1]. The primary role of this enzyme is considered to be the elimination of harmful microorganisms through the process of phagocytosis by neutrophils [2,3]. It has robust antibacterial characteristics and can produce highly effective bactericidal chemicals, such as hypochlorous acid (HOCl). It plays a crucial part in the eradication of parasites, fungi, protozoa, viruses, and tumor cells [1,4]. Therefore, individuals with MPO deficiency have a significantly increased incidence of serious infections and chronic inflammatory diseases [1,4]. However, MPO deficiency may also confer certain benefits, an area currently under extensive investigation. For example, it may provide protection against cardiovascular damage, inhibit the progression of chronic kidney disease, and mitigate skin injury caused by acute inflammatory responses [5].

MPO deficiency, first documented in 1954, is a genetic condition characterized by autosomal recessive inheritance and caused by mutations in the MPO gene on chromosome 17q22-23 [1,6]. A secondary MPO deficiency may result from somatic mutations of the MPO gene; it is less frequent than the hereditary form. Another form, acquired, is often temporary and resolves as the underlying condition improves. MPO-deficient patients have impaired microbial elimination but are typically asymptomatic, with the exception of diabetics [1].

## Case presentation

A patient, a 37-year-old Iranian woman from Tabriz, presented to the outpatient clinic of Shahid Ghazi Hospital with a chronic history of recurrent bacterial and fungal infections dating back to childhood. These infections have infected her from early childhood and have persisted throughout her life. She does not report any other significant medical conditions beyond these recurring infections. Notably, there is no familial history of similar diseases, and there are no reports of similar conditions in other family members. The patient also indicated a chronic history of using antibiotics and antifungal medications, which has significantly impacted her quality of life.

A comprehensive clinical assessment of the patient revealed a generally stable and well-appearing individual without any overt health concerns. There were no indications of neurological symptoms, and no cutaneous manifestations were noted. Furthermore, the patient's vital signs, including heart rate, blood pressure, and body temperature, were all within the normal range during the examination.

Given the patient's recurrent infections without other obvious signs of illness, a complete blood count (CBC) with a differential white blood cell (WBC) count was requested. The laboratory results upon admission are shown in Table 1.

**Table 1:** CBC CBC: complete blood count; WBC: white blood cell count; RBC: red blood cell count; HBG: hemoglobin; HCT: hematocrit; MCV: mean corpuscular volume; MCH: mean corpuscular hemoglobin; MCHC: mean corpuscular hemoglobin concentration; RDW: red cell distribution width; PLT: platelet count; ml: microliter; g/dL: grams per deciliter; fL: femtoliter; pg: picograms; %: percent

	Number of cells	Reference
WBC (*10^3^/ml)	4.81	3.8-10.4
RBC (*10^6^/ml)	5.18	3.8-5
HBG (g/dL)	14.6	11.9-14.8
HCT (%)	42.5	35.9-44.6
MCV (fL)	82.1	82.5-98
MCH (pg)	28.1	26-33
MCHC (g/dL)	34.3	32.5-35.2
RDW (%)	13.5	11.4-13.5
PLT (*10^3^/ml)	166	153-361
Neutrophils (*10^3^/ml)	0.23	2500-7000
Lymphocytes (*10^3^/ml)	2.16	1000-4800
Monocytes (*10^3^/ml)	1.98	200-800
Eosinophils (*10^3^/ml)	0.09	30-350
Basophils (*10^3^/ml)	0.06	Up to 300

**Figure 1:**
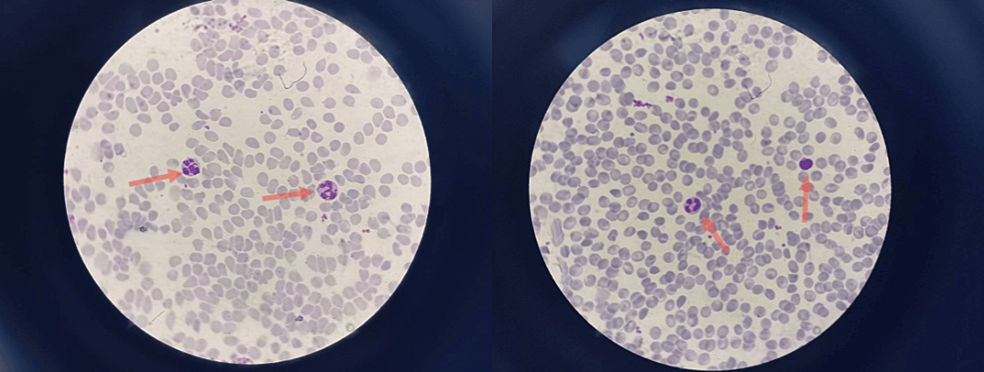
PBS Figure 1 revealing reduced neutrophil granulation and abnormal neutrophil morphology (red arrows) PBS: peripheral blood smear

Upon observing the decreased number of neutrophils despite normal WBC counts, a peripheral blood smear (PBS) was performed. It revealed reduced neutrophil granulation, abnormal neutrophil morphology, decreased chromatin condensation, and cytoplasmic hypogranulation (Figure 1).

Immunocytochemistry, or, more frequently, flow cytometry, is often employed to diagnose MPO deficiency by assessing functional MPO levels within neutrophils [1]. However, due to the high cost of this test, the patient opted not to undergo flow cytometry to reduce additional expenses. Consequently, we decided not to proceed with ordering this test in this case.

Based on the clinical history, laboratory findings (CBC H1), and PBS, the patient is diagnosed with MPO deficiency. As the patient had no symptoms at the time of diagnosis, no immediate action was taken. The recommended follow-up for this patient involves active surveillance, with instructions to monitor for warning signs such as flu-like symptoms, urinary tract infection symptoms, and skin infection symptoms. If any of these symptoms occur, the patient should promptly seek medical attention for further evaluation and appropriate treatment. During this period, the patient has been visiting every three months with a CBC check-up.

Differentiating MPO deficiency from conditions with comparable clinical manifestations involves distinguishing it from disorders like chronic granulomatous disease (CGD), Chediak-Higashi syndrome, leukocyte adhesion deficiency, and any underlying condition capable of inducing secondary MPO deficiency [1]. In cases of disseminated fungal infections, MPO deficiency should be included in the list of potential differentials. To exclude these differential diagnoses, it is important to note that patients with CGD typically present with fever, malaise, weight loss, and persistent perirectal abscesses and can be diagnosed using the nitroblue tetrazolium (NBT) reduction rest [7]. Chediak-Higashi syndrome is identified through microscopic examination of blood smears revealing giant lysosomal granules in leukocytes, alongside partial albinism and neurological symptoms [8]. Leukocyte adhesion deficiency is diagnosed by delayed separation of the umbilical cord, absence of pus, and recurrent skin infections [9]. This case does not exhibit these symptoms, and we were able to rule out these syndromes, despite their common characteristic of recurrent pyogenic infections based on phagocytosis and neutrophil dysfunction.

## Discussion

This patient presented with a substantial health challenge due to her lifelong chronic and recurrent fungal and bacterial infections, which commenced in her childhood. Although the infections remained recurrent and did not manifest any obvious clinical symptoms, they significantly affected her general health and quality of life.

Significantly, there was no genetic history of such illnesses, and other relatives did not report similar symptoms. The lack of familial medical records prompted inquiries on the hereditary pattern and the exceptional nature of the patient's condition [1,6]. Additionally, the lack of neurological symptoms, cutaneous indications, or vital sign abnormalities during clinical examination complicates the diagnosis procedure even more. This unusual appearance is typical of MPO deficiency, which is frequently asymptomatic in individuals unless there are specific cases such as diabetes [1,10].

The laboratory assessment, specifically the differential WBC count as part of the CBC, was instrumental in identifying the fundamental immunological anomaly. The hematological analysis described here is crucial for determining the function and composition of different categories of blood cells. Furthermore, the patient's CBC revealed a reduction in neutrophils, an essential constituent of the primary immune defense mechanism against fungal and bacterial infections [1]. Despite a normal WBC count, this neutropenia was a crucial indicator of immunological dysfunction. The choice to do a PBS helped to confirm the diagnostic hypothesis. Neutrophils in PBS showed a number of problems, such as less granulation, abnormal morphology, less chromatin condensation, and hypogranulation in the cytoplasm. These distinguishing traits are consistent with the results of MPO deficiency [2]. Definitive diagnosis is determined by histochemical staining of neutrophils for MPO, accessible in commercial laboratories.

The diagnosis of MPO deficiency is crucial for controlling the patient's recurring infections. MPO-deficient people may not present overt symptoms of the illness as asymptomatic carriers, making early diagnosis by hematological investigation critical. It is crucial to establish and execute a comprehensive care strategy that prioritizes consistent surveillance for indications of infection such as *Candida* strains, hyphal forms of *Aspergillus fumigatus*, and *Mycobacterium tuberculosis* [1,2,11] and the development of diseases (e.g., diabetes mellitus, polyarthritis, autoimmune lupus nephritis, etc.) due to its role as an anti-inflammatory mediator [1,4,12]. Also, it is better to mention that genetic counseling may be incorporated into management strategies, especially in cases where the patient is contemplating family planning.

This case report has limitations due to the patient's unwillingness to undergo costly tests. The first test, flow cytometry, is often used to diagnose MPO deficiency [1]. The second test, the NBT reduction test, is used to rule out CGD [7]. However, the patient has been consistently following up with CBC check-ups every three months. Based on the PBS, CBC sequence, symptoms, and patient history, other diagnoses have been ruled out, confirming the diagnosis of MPO deficiency.

## Conclusions

MPO deficiency is a rare condition often characterized by a lifelong history of recurrent infections, although most patients remain asymptomatic except for those with diabetes. Confirming the diagnosis requires thorough hematological analysis, PBS, and flow cytometry. This case underscores the importance of heightened clinical awareness and comprehensive diagnostic approaches in effectively identifying and managing MPO deficiency, ultimately improving patient outcomes and quality of life.
